# Non-traumatic Atlantoaxial Subluxation Following Pharyngitis in Healthy Children: A Case Series

**DOI:** 10.7759/cureus.93005

**Published:** 2025-09-23

**Authors:** Shinas K Nellicka, Amani A Azizalrahman, Irshad Abdulmajeed

**Affiliations:** 1 Pediatric Emergency Medicine, King Fahad Medical City, Riyadh, SAU

**Keywords:** atlantoaxial instability, atlantoaxial subluxation, atraumatic, grisel’s syndrome, ligamentous laxity

## Abstract

Grisel syndrome is a rare condition characterized by non-traumatic rotary subluxation of the atlantoaxial joint. It is commonly seen in pediatric patients, but can also be seen in adults. Although rare and with only a few reported cases, a high index of suspicion is required for prompt recognition and early treatment of this condition, as it is essential in preventing permanent deformity and neurological deficits. We report two pediatric cases; the first was a 13-year-old girl presenting to the pediatric emergency department with neck pain and head tilt for two weeks. She had a history of pharyngitis but no trauma. On examination, there was neck pain on movement with torticollis but no midline tenderness. CT confirmed Atlantoaxial subluxation. The second case was a three-year-old boy who presented with an acute onset of neck pain. He had a history of fever with throat pain one week before presentation, and no history of trauma. On examination, there was neck pain on movement with torticollis. CT of the cervical spine in both cases showed evidence of Atlantoaxial subluxation. The first case was offered conservative management with excellent clinical improvement and complete resolution of symptoms and signs. However, the second case was offered conservative management, antibiotics, and analgesia, but unfortunately, no prognosis was known for this patient as he was lost to follow-up. Children presenting with torticollis with a recent history of fever or pharyngitis and without a history of trauma should raise suspicion of Grisel's syndrome. Surgical intervention is indicated only in cases of high-grade instability. Early diagnosis allows for treatment without the need for surgery in most patients; surgical intervention is only indicated in the presence of severe instability.

## Introduction

Grisel syndrome is a rare, non-traumatic atlantoaxial subluxation, typically seen in children, though cases in adults have been reported. This condition usually arises after an upper respiratory tract infection or otolaryngologic procedure, with the underlying mechanism believed to involve hyperemia of the pharyngo-vertebral venous plexus, leading to ligamentous laxity between the atlas (C1) and axis (C2) vertebrae [1]. The proximity of these venous systems explains the connection between head and neck infections and the development of atlantoaxial instability.

Though Grisel syndrome is rare, its incidence may be underreported due to its nonspecific clinical presentation, often manifesting as torticollis or neck stiffness in the absence of trauma. In children, the condition is typically triggered by infections such as pharyngitis, tonsillitis, or otitis media, with Streptococcus being one of the more common pathogens identified [2,3]. Early recognition is crucial because delayed or missed diagnosis can lead to progressive deformity and, in severe cases, permanent neurological damage due to spinal cord compression.

Diagnosis of Grisel syndrome requires a high index of suspicion, especially in children with a history of recent ear, nose, or throat infections or neck pain. Radiological studies, including CT and MRI, play a pivotal role in confirming the diagnosis [4]. CT scans can reveal atlantoaxial subluxation, while MRI may help assess any associated soft tissue involvement or spinal cord compression.

Management of Grisel syndrome varies according to the severity of the subluxation. Conservative treatment, including immobilization and anti-inflammatory therapy, is effective in most mild cases. However, severe or persistent subluxation may require surgical intervention, particularly in cases with significant instability or neurological symptoms. Although rare and based on only a few reported cases, early diagnosis and appropriate management are essential to prevent long-term complications and reduce the need for invasive treatments [3].

## Case presentation

Case one

**Figure 1:**
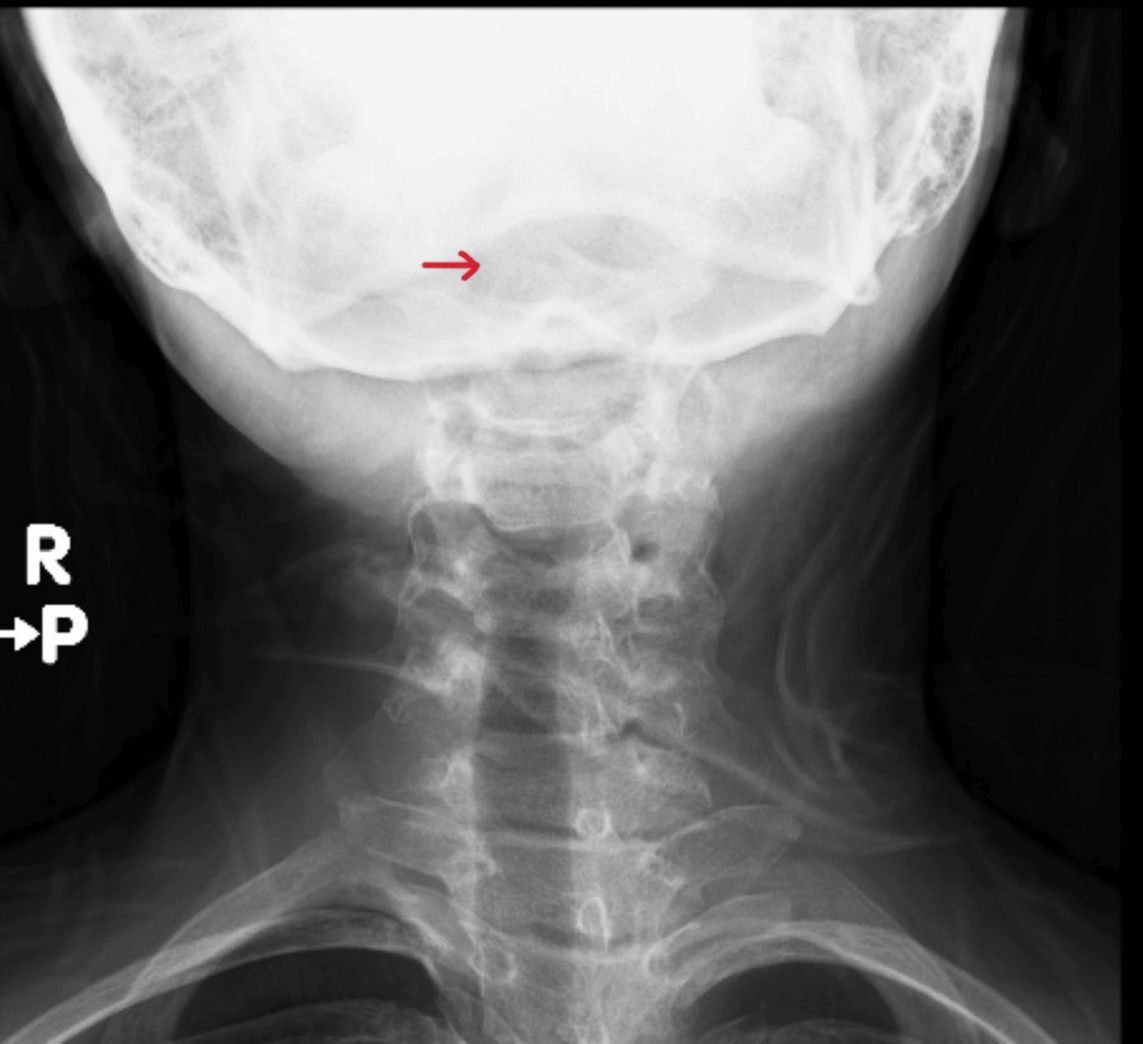
X ray showing torticollis with suspicion of cervical spine injury

**Figure 2:**
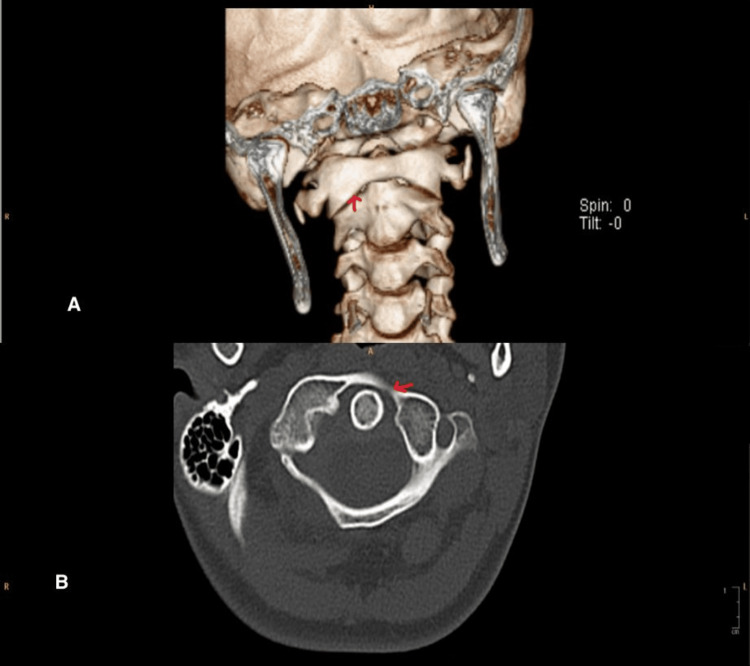
Atlantoaxial subluxation showing in the 3-Dimensional and a axial view of a CT cervical spine A: 3-dimensional view; B: the axial view.

**Figure 3:**
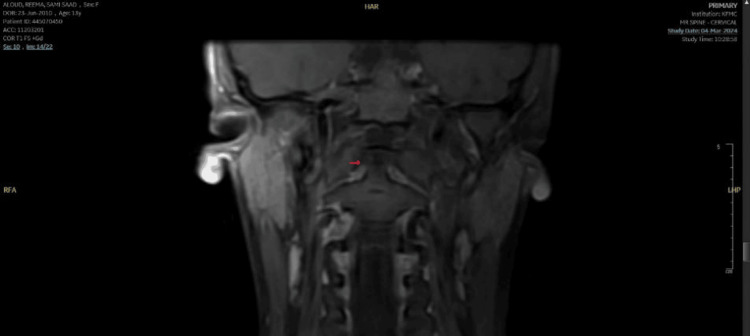
MRI coronal view of a cervical spine showing resolution of AAS AAS: atlantoaxial dislocation

A 13-year-old previously healthy girl presented to our pediatric emergency department with neck pain and head tilt for two weeks. It was sudden in onset after getting up from sleep. She had a history of pharyngitis at the onset, which resolved in seven days. There was no clear history of trauma. There is no history of fever, upper respiratory infection-related symptoms, or joint pain. No family history of rheumatological diseases. Initially, the patient was managed with analgesics from the primary health clinic, and mild improvement was observed. On examination, she was a vitally stable, well-looking child with no apparent dysmorphic features but had obvious torticollis with no significant midline cervical tenderness or neurological deficits. Other systemic examinations, including musculoskeletal examinations, were within normal limits. Given persisting torticollis, X-ray (Figure 1) was done, raising suspicion of atlantoaxial subluxation (AAS), following which CT was done (Figure 2), which confirmed AAS. Labs showed normal cell counts but raised inflammatory markers. A pediatric spine surgeon was involved, advised for conservative management, neck immobilization with a soft collar, and analgesia. The child had clinical improvement within three weeks. A follow-up MRI (Figure 3) showed complete radiological resolution.

Case two

**Figure 4:**
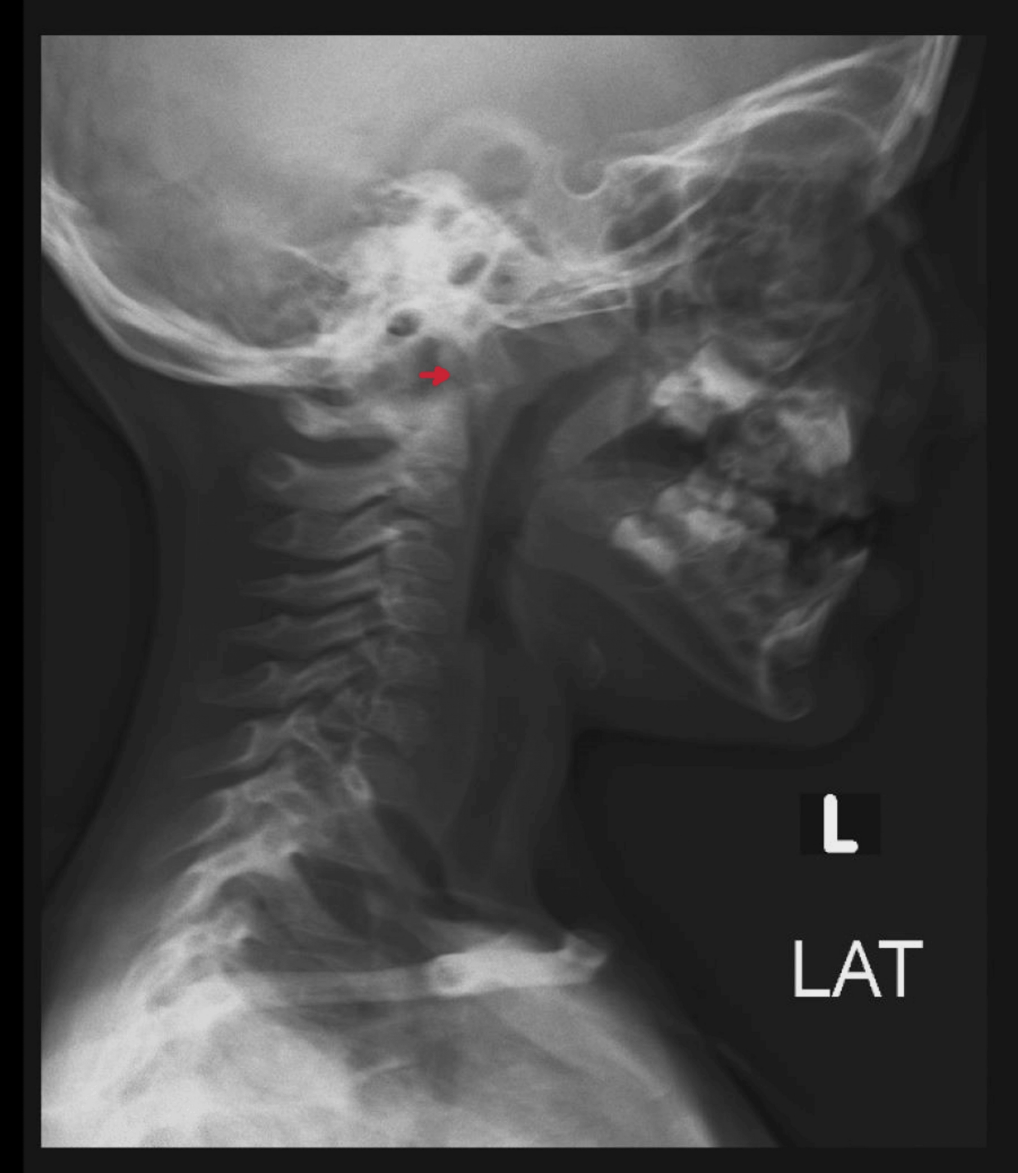
X ray showing suspicion for atlantoaxial subluxation

**Figure 5:**
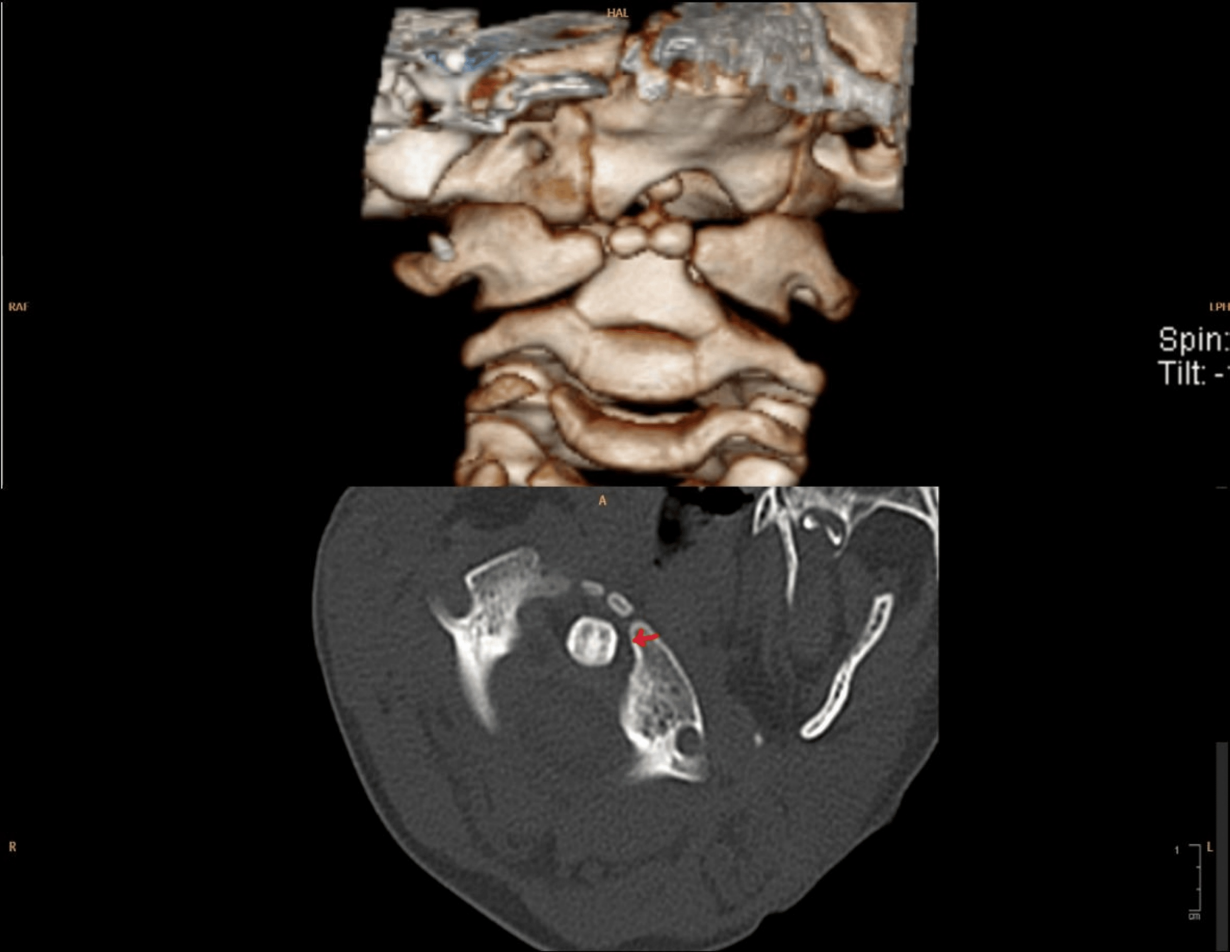
Atlantoaxial subluxation showing in the 3-Dimensional and a axial view of a CT cervical spine A: 3-dimensional view; B: the axial view.

A three-year-old boy, previously healthy, presented to the pediatric emergency department with an acute onset of neck pain. The child had a history of fever with throat pain one week back and was treated with oral antibiotics from another healthcare facility. There is no history of trauma, fever, or throat pain on presentation. The child was vitally stable with no apparent dysmorphic features. On further examination, there was neck pain and mild torticollis on movement. He had no midline cervical tenderness or any neurological deficits. Other systemic examinations, including a complete musculoskeletal examination, were within normal limits, and the child did not have any laxity of ligaments. X-ray (Figure 4) of the cervical spine, including an open mouth view, was done, which was suspicious, and a CT of the cervical spine (Figure 5) showed evidence of AAS. Laboratory values were unremarkable apart from high inflammatory markers. Spine surgery was consulted, and the patient was discharged with antibiotics, analgesics, and a soft collar. The patient was lost to follow-up, so the prognosis is unknown.

## Discussion

Grisel syndrome is a condition of unknown etiology characterized by atraumatic atlantoaxial subluxation due to inflammatory ligamentous laxity following an infectious process in the head and neck. Though a relatively rare condition, it may lead to severe consequences with Neurological complications ranging from radiculopathy to death from medullary compression [2]. Sir Charles Bell reported the first case in 1830 after atlantoaxial subluxation resulted in spinal cord compression in a patient with syphilitic ulceration of the pharynx [5]. A French physician, Grisel, described two cases of pharyngitis and non-traumatic AAS and named it Grisel syndrome [6]. Grisel syndrome primarily affects the pediatric population, with 68% of cases occurring in patients under the age of 12 years [7] and 90% under the age of 21 [8]. Although the etiology is largely unknown, Battiata et al. [9] proposed the two-hit hypothesis that the first "hit" is a pre-existing cervical ligamentous laxity seen in the pediatric population at baseline. The second "hit" is the cervical muscle spasm caused by the inflammatory mediators transported by the pharyngeal vertebral plexus. Early detection of atlantoaxial subluxation and adequate antibiotics and anti-inflammatory therapy can lead to early recovery. Deichmueller and Welkoborsky[10] reported that four of 12 patients who started therapy with Grisel syndrome had persistent torticollis despite adequate conservative treatment, and those patients required a delayed external fixation. Conventional X-rays of the cervical spine, including an open-mouth view, can be helpful in identifying this condition, but CT is usually required and may prove more useful. The primary treatment of early-detected Grisel syndrome is conservative, including neck support, anti-inflammatory drugs, and, in some cases, antibiotics. Thus, as we have seen in our two cases, there is an increased need for awareness amongst Pediatric emergency physicians regarding Grisel syndrome, and a high index of suspicion is needed for early diagnosis and prompt intervention.

## Conclusions

Grisel Syndrome is an uncommon entity in the pediatric population with severe consequences if timely diagnosis and management are not initiated. Therefore, a high index of suspicion is required for early diagnosis. Due to the radiation risk, X-ray and CT scans are usually avoided in children; hence, our case report will further help emergency pediatricians decide. Both our cases had no apparent history of trauma. They were not responding to symptomatic treatment, prompting us to investigate further. Our cases were diagnosed early and given prompt treatment, leading to full recovery in one of the patients, but unfortunately, the second patient was lost to follow-up.
